# Health Care Utilisation by Bullying Victims: A Cross-Sectional Study of A 9-Year-Old Cohort in Ireland

**DOI:** 10.3390/healthcare6010019

**Published:** 2018-02-25

**Authors:** Catherine Hayes, Dervla Kelly, Cristina Taut, Elizabeth Nixon, Lina Zgaga, James Williams, Thomas O’Dowd, Udo Reulbach

**Affiliations:** 1 Department of Public Health and Primary Care, Trinity College Dublin, Dublin, Ireland; dkelly23@tcd.ie (D.K.); tautc@tcd.ie (C.T.); ZGAGAL@tcd.ie (L.Z.); todowd@tcd.ie (T.O.); udo.reulbach@gmail.com (U.R.); 2 School of Psychology Trinity College Dublin, Dublin, Ireland; enixon@tcd.ie; 3 Economic & Social Research Institute (ESRI), Dublin, Ireland; james.williams@esri.ie; 4 Department of Child & Adolescent Psychiatry, Our Lady's Children's Hospital Crumlin, Dublin, Ireland

**Keywords:** bullying, gender, primary health care, general practice, health care utilisation, mental health

## Abstract

Children frequently refrain from disclosing being bullied. Early identification of bullying by healthcare professionals in children may prevent adverse health consequences. The aim of our study was to determine whether Health Care Utilisation (HCU) is higher in 9-year-olds who report being bullied and factors influencing type of HCU. The study consists of cross-sectional surveys of Child Cohort of Irish National Longitudinal Study of Children (Wave 1), 8,568 9-year-olds, and their carers. Being bullied was assessed by a self-reported questionnaire completed by children at home. HCU outcomes consisted of the following: visits to GP, Mental Health Practitioner (MHP), Emergency Department (ED), and nights in hospital by parent interview. Bivariate logistic regression and gender-stratified Poisson models were used to determine association. Victimisation by bullying independently increased visits to GP (OR 1.13, 95% confidence interval (CI): 1.03 to 1.25; *p* = 0.02), MHP (OR 1.31, 95% CI: 1.05 to 1.63; *p* = 0.02), though not ED visits (OR 0.99, 95% CI: 0.87 to 1.13; *p* = 0.8) or nights in hospital (OR 1.07 95% CI: 0.97 to 1.18; *p* = 0.2), adjusting for underlying chronic condition(s) and socio-demographic confounders. Victimised girls made higher GP visits (RR 1.14, 95% CI: 1.06 to 1.23; *p* < 0.001) and spent more nights in hospital (RR 1.10, 95% CI: 1.04 to 1.15; *p* < 0.001). Victimised boys were more likely to contact MHPs (RR 1.21, 95% CI: 1.02 to 1.44; *p* = 0.03). 9-year-old bullied subjects were more likely to utilise primary care services than non-bullied 9-year-olds. Different HCU patterns were observed according to gender and gender differences in the presentation of victimisation. Our findings may lead to the development of clinical practice guidelines for early detection and appropriate management of bullied children.

## 1. Introduction

Bullying may be defined as a recurrent, intentional, and unprovoked form of aggressive behaviour to inflict pain on, or cause distress to, another individual [1,2,3]. Bullying may be subcategorised as direct, e.g., physical or verbal, or indirect or relational bullying, e.g., social isolation or cyber bullying. The prevalence of bullying and being bullied in early and late adolescence varies widely depending on the definition of bullying used, age, gender, social class, and the country being assessed [2,3,4]. This study concerns children who were victims of bullying. The European Health Behaviour in School Children (HBSC) survey [4] of data collected between 2013–2014 has shown that 9–45% of 11-year-old boys and 5–36% of 11-year-old girls self-reported being bullied at least twice in the few months preceding the survey. The 2014 Irish HBSC survey of children aged 8.5–10.5 years reports 36% of children “ever being bullied at school once in the past few months” [5], which is virtually unchanged from 2006 (37%) [6] and 2010 (37%) [7]. Significant gender or social class differences were not observed in this younger age cohort, unlike in older children [5]. 

Whether being bullied per se is associated with utilisation of health services is an under-researched area and the direction of any potential association is unknown. It is well recognised that children often hold back on disclosing bully victimisation, which represents a major barrier to its resolution [8]; this may suggest an association with decreased attendance. On the other hand, the presence of chronic mental or physical illhealth confounds the relationship between being bullied and health services utilisation. A two-way cause and effect relationship between the victim of bullying and having an underlying chronic physical or mental health condition has been addressed in several papers [9,10,11]. Not only can victimisation lead to deterioration in health, which results in increased primary and secondary health care utilisation, but children with a chronic condition(s) are also more likely to be victimised by bullying [9,11,12].

Research has found that psychosomatic disturbances are among the most frequent mental health problems in children presenting to general practitioners and other primary care professionals, and that interventions to address these mostly consist of parental counselling [13]. While the consequences of bully victimisation on mental health have been widely explored, research is sparse on the presentation and use of health services by children, especially younger children, who are bullied [14]. A survey of changes in mental health, bullying behavior, and service use among approximately 1000 8 year olds in Finland over 24 years (1989 to 2013) showed that involvement in bullying (bullies, victims, and bully victims) was strongly associated with mental health problems [15].

The relationship between disability among children and exposure to bullying is an under researched area. There is no consensus on how to define disability, but children categorised as disabled are more often subjected to victimization compared to non-disabled children [12,16,17]. Fridh et al. (2017) found disabled adolescents report poorer health and are more exposed to both traditional bullying and cyber harassment [17]. Secondary analysis of other European and Australian general population cohort studies involving younger children also report higher and more persistent rates of mental health and behavioural issues with children with intellectual disabilities compared to their peers without intellectual disabilities [18,19,20]. 

Whether or not there are any differences in being bullied in childhood, by gender, or type of health service sought is also unknown. In the general literature, few studies have examined the association between gender and health service use among children, and findings from these studies are mixed. Flisher et al. [21] found no relationship between gender and requiring mental health services during childhood. Cuffe et al. [22] described higher health service usage among boys in early adolescence and among girls in later adolescence. A U.S. study of 274 14–15 year olds reported that more girls than boys turned to a friend for help with an emotional concern, and girls were twice as likely as boys to report willingness to use mental health services (odds ratio 2.45, 95% confidence interval (CI) 1.20 to 4.99) [23]. Garland et al. [24] reported that males aged 10-13 years were much less likely than females to seek help from adults for a psychosocial problem. 

Arising from the literature above, we hypothesised that younger children who are victims of bullying may be more likely to utilise health care services than non-victimised children; however, this may be confounded by presence of chronic illness/disability [12,16,17]. Our study hypothesis therefore tested (1) whether there is an associated increase in the utilisation of primary and secondary care health services in 9-year-old children who are victims of bullying, independent of the presence of chronic illness and/or disability; and (2) whether gender differences in the type of health care utilised by victims of bullying existed.

## 2. Materials and Methods 

### 2.1. Study Design

Data were provided from the first wave of data from the Child (9-year-old) cohort of the Irish National Longitudinal Study of Children, Growing Up in Ireland (GUI). Inclusion in the study was on an opt-in basis with consent and assent forms signed by parent(s)/guardian(s) and study child.

Participants were selected using a two-stage sampling method [25,26]. In stage one, 1105 primary schools from a national total of 3200 were randomly selected using probability proportionate to size (PPS) sampling [25], of which 910 primary schools participated (response rate, 82%). The schools provided the sampling frame for stage two. The final Growing up in Ireland cohort sample size comprised 8568 children (4404 girls and 4164 boys), or approximately 1 in 7 (14%) of resident 9-year-olds [27]. A subset of 8183 children was used in our study, as those with missing information on being bullied in either self or parent questionnaire were excluded. 

The study design was approved by the Scientific and Policy Advisory and the Research Ethics Committees of the Irish Health Research Board. Questionnaires were designed by a study team using a Delphi process involving a wide range of stakeholders. Questionnaires were piloted firstly with a Children’s Advisory Forum (84 children) and then with a random sample of 145 families recruited via nine randomly selected schools (response rate 136/145, 94%). A second pilot was conducted with a further 62 children and their families. Minor modifications were made to the questionnaires as a result of pilot testing.

### 2.2. Survey Design and Data Collection

Study participants were recruited between September 2007 and June 2008. Bully victimisation data were obtained from directly from children who completed a paper Child Sensitive Questionnaire (CSQ) provided by the interviewer on their own at home. The CSQ was designed with input from children themselves and included pre-pilot and pilot studies to test validity, which are described in more detail elsewhere [26].

The child was asked: “*Thinking back over the last year would you say that anyone (either a child or an adult) picked on you*”. A positive answer was followed by a prompt to specify the forms of bullying experienced; shoving, pushing, hitting, name calling, slagging (teasing), text messaging, emails, written messages/notes, cyber bullying, deliberate exclusion from games/conversations, or other forms of bullying. 

In addition to the child survey, a survey was carried out with the primary care givers by trained interviewers. Pre-defined healthcare utilisation (HCU) measures of frequency of contact with primary health care professionals were obtained (adpated from the Canadian National Longitudinal survey of Children and Youth): number of contacts with GP, another medical doctor or mental health professional (MHP), i.e., psychologist, psychiatrist, or counsellor over the previous 12 months. Child’s use of hospital and Emergency Department visits—number of nights the child spent in hospital over his/her lifetime (excluding time of birth) and number of visits to ED over the previous 12 months—were sought as an indicator of severe morbidity. Parent report of bullying prevalence was also sought in an interview question: “Has (*insert name of Study Child*) been a victim of bullying in the past year”.

The following information was sought on potential confounders; child factors included gender, presence/absence of chronic condition, and measured BMI; parental/family factors included parental age household composition, education level, household occupational class, general medical services entitlement, and social welfare entitlement [28]. 

The primary caregiver was asked whether or not the study child had a chronic condition: defined as “any on-going chronic physical or mental health problem, illness or disability?” If this question was answered in the affirmative, an open-ended question was asked: “What is the nature of this problem, illness or disability? Please describe as fully as possible.” Responses to this item were coded using International Classification of Diseases 10 [29].

The primary caregiver was also asked about specific learning disability (LD), communication, or coordination disorders. For ease of interpretation, all subsequent references to “ongoing chronic condition” refer to ongoing chronic illness or disability, including LD, unless specifically mentioned.

### 2.3. Statistical Analyses

All results are based on statistically reweighted data to ensure representation of all 9-year-olds in Ireland and to account for the complex sampling design. Specific details of sampling frames and methodology, weighting strategies, questionnaires, and response rates have been reported elsewhere [26].

Chi-Square tests summarised the proportion of children being victimised and type of bullying experienced, and tested gender differences in victimisation prevalence. Multivariable logistic regression models were used to determine association between HCU and bully victimisation: Any visits to GP vs. None; Any visits to MHP vs. None; Any visits to ED vs. None; Nights in Hospital vs. None. Confounding variables were selected using a backwards stepwise method. Odds Ratios (OR) with 95% CIs described the strength of the association. Gender stratified Poisson models examined specific associations by gender. All models were adjusted for potential confounders. The cut-off of the *p*-value for statistical significance was 0.05. Analysis was carried out in SPSS V24.

The STROBE checklist for reporting of cross-sectional studies was followed in the writing of this article [30].

## 3. Results

Table 1 summarises the proportion of the total cohort who experienced bully victimisation in the past year according to self and parent report, the form(s) of bullying experienced, and breakdown by gender. Four in 10 children reported being a victim of bullying, with no gender difference in prevalence (χ_2_ = 0.09; *p* = 0.8). Boys were more likely to report being physically (χ_2_ = 157.1; *p* < 0.001) or verbally bullied (χ_2_ = 41.3; *p* < 0.001). Girls were more likely to report being bullied by exclusion (χ_2_ = 23.0; *p* < 0.001) and by written messages (χ_2_ = 4.37; *p* = 0.04). While 39.9% of the study children disclosed having been bullied, 23.5% of parents thought their child was/had been bullied (Table 1). All data from Table 2 onwards refer to child-reported bullying.

Table 2 summarises the healthcare utilisation, child health and socio-demographic characteristics of bullied and non-bullied subjects in our cohort on univariate analysis. Results demonstrated significantly higher GP use and contact with Mental Health Professionals (MHP) among bullied subjects.

The logistic regression model comparing “Any visits” with “No visits”(Table 3) for each type of HCU showed that victimised children were 13% more likely to visit GPs (OR 1.13, 95% CI:1.03 to 1.25, *p* = 0.02) and 31% more likely to visit MHPs (OR 1.31, 95% CI: 1.05 to 1.63; *p* = 0.02), but were not likely to visit EDs (OR 0.99, 95% CI: 0.87to 1.13; *p* = 0.8) or spend nights in hospital (OR 1.07; 95% CI: 0.97 to 1.18; *p* = 0.18), following adjustment for ongoing chronic condition and socio-demographic factors. 

Other factors associated with increased HCU irrespective of bullying status in the bivariate model were presence of chronic illness (higher GP and ED visits), learning disability (higher GP and ED visits), full entitlement to free medical care (higher GP visits), and children of lone parent families with a sibling (higher MHP visits). Children of parents with lower educational attainment were less likely to visit GPs and MHPs (Table 3).

Victimised boys were less likely to attend GPs than girls (OR 0.84; 95% CI: 0.76 to 0.93; *p* = 0.001). Although victimised boys were more likely to seek MHP assistance than girls (OR 1.25; 95% CI: 0.99 to 1.56), this was not statistically significant in the regression model (*p* = 0.057) (Table 3). In summary, Table 3 showed the increased likelihood of having visited a professional among victimised children compared to non-victimised children, adjusting for confounding personal and socioeconomic characteristics.

From the Poisson models (Table 4), bully victimisation was confirmed as a highly significant independent risk factor for GP visits (Incident Rate Ratio (IRR) 1.11, 95% CI:1.05 to1.16; *p* < 0.001)) and for MHP visits (IRR 1.15, 95% CI: 1.01 to 1.32; *p* = 0.04), (Model 1). The relationship was attenuated (IRR 1.13, 95% CI: 0.99 to 1.29; *p* = 0.07) when MHP visits were also adjusted for GP visits (Model 2). Unlike the logistic model, the Poisson model demonstrated an association between being victimised and nights in hospital (IRR 1.08, 95% CI: 1.04 to 1.11; *p* < 0.001) (Model 1). The relationship was only slightly attenuated when nights spent in hospital were adjusted for GP and MHP visits (Model 2). The Poisson model confirmed lack of association between bully victimisation and ED attendance [31]. 

Multivariable analysis found higher GP visits by victimised girls (IRR 1.14, 95% CI: 1.06 to 1.23; *p* < 0.001). Poisson models showed victimised girls spent more nights in hospital having adjusted for GP and MHP visits and other confounders (IRR: 1.10, 95% CI: 1.04-1.15, *p* < 0.001), not observed for victimised boys (Table 3). Unlike the multivariable model, the fully adjusted Poisson model confirmed that victimised boys were more likely to contact MHPs (IRR 1.21, 95% CI: 1.02 to 1.44; *p* = 0.03) but not girls (IRR 1.02, 95% CI: 0.82 to 1.26; *p* = 0.88) (Table 4). Table 4 summarised the higher number of visits among victims compared to non-victims, adjusting for confounding personal and socioeconomic characteristics.

## 4. Discussion

### 4.1. Summary

Our study sought to determine an association between being bullied as a 9-year-old and frequency of primary health service attendance within a 12 month period and ever use of secondary healthcare. It sought to establish if HCU is higher in 9-year-olds who report being bullied compared to non-victims and factors influencing HCU. Our results suggest that self-reported bully victimisation is associated with higher HCU, irrespective of having a pre-existing chronic condition, socio-demographic background, or free GP care. Although gender differences in forms of bullying experienced are well documented, our study also found significant differences in type of health service used, with girls more likely to present to GPs and spend nights in hospital, and boys more likely to present to MHPs. 

### 4.2. Strengths and Limitations

A major strength of this study is the large national representative sample, which equates to approximately 1/7 of all 1997 births in Ireland [26,32]. The CSQ is likely to have improved the extent and accuracy of reporting, as it was completed privately by children in their own homes. We have adjusted our analysis of HCU for a wide range of confounders associated with exposure (bullying) and outcome (HCU).

Our analysis is limited in that data are self-reported, which, though it may facilitate disclosure, may also bias responses of 9-year-olds depending on their understanding of the questions asked. However, the CSQ was developed with input from children themselves and piloted extensively to address this issue. With regard to the definition of bullying used, the child was asked to “think back over the past year” in relation to occurrence of being bullied which implied a “period prevalence” rather than a “point prevalence” of victimisation. The prompts were given to elicit the form of bullying experienced, e.g., hitting, name-calling, deliberate exclusion, and conveying deliberate intention. However, data on the number of bullying episodes over the past year were not sought, which is a limitation of the study. In addition, data were not available on severity and intensity of victimisation that may have elicited a more nuanced understanding of HCU according to bullying experiences. However, difficulty in obtaining this type and quantity of data from a 9-year-old should not be underestimated. It will not encourage all young people who are being bullied to disclose and is still likely to be subject to an under-reporting bias. However, prevalence rates from child self-report were much higher than parent-reported rates. The principal limitation of the study relates to the cross-sectional nature of the data, which precludes determination of causal relationships between bullying and HCU; hence, our findings are limited to association. 

### 4.3. Comparison with Existing Literature

The negative impact of bullying on children’s health has been well researched, particularly in those with underlying chronic illness or disability [33]. It is therefore unsurprising that victimisation is associated with HCU and, especially, GP utilisation. Furthermore, having a long-term condition or LD may contribute not only to HCU but also to the person being bullied in the first place. 

Many children who see a MHP and/or spent nights in hospital will have been referred via general practice. Gender differences in GP attendance by adolescents with depressive symptoms have been noted previously [34]. Adolescent boys were reported to find it difficult to discuss psychological problems with GPs but were more likely to attend to physical problems. Female attendance was predicted in part by a belief that the GP’s role included an interest in psychological symptoms. Vila et al. reported that, in addition to physical health problems, social factors and psychiatric difficulty are significant in young people who frequently attend primary care [35]. 

Several theories have emerged to explain gender differences in health resource use in different contexts, biological and/or behavioural reasons, and bias in the referral process [36]. It may be that distress associated with victimisation manifests differently in boys and girls. Girls may be more likely to present with physical manifestations of psychosomatic symptoms and boys with behavioural problems, thus leading to perceived differences in healthcare needs. The higher rates of physical and verbal bullying experienced, and higher prevalence of LD, are likely to contribute to greater contact with MHPs by boys. Previous studies suggest that boys with behavioural difficulties attract most attention and resources [37,38]. Hartley et al. found that students in special education reported higher levels of physical victimisation, as well as more physical and emotional harm in addition to increased psychological distress as a result of their victimisation [39].

### 4.4. Implications for Research and/or Practice

Previous studies indicate that families are often unaware of the child’s victimisation due to a reluctance to disclose [8]. Our study confirms a gap between parents’ perception of their child being bullied and a child’s self-reported experience of victimisation [40]. How parental knowledge and engagement in recognising bullying may be improved is a subject for further research. Although the dataset was published in 2008, there have been negligible public health interventions targeting bullying, so we believe the data is still relevant to the current experience of children. Parents, teachers, and healthcare professionals all need to be aware of the high prevalence of victimisation in children and the different ways that this may manifest. Regarding health professionals, there has been some preliminary work on the acceptability of routine questioning by clinicians, which found over half of young people would be happy for their GP to ask about bullying, and over 80% would be happy to complete a screening questionnaire before seeing their GP [41]. This mirrors the UK’s National Society for the Prevention of Cruelty to Children report, which found that young people wanted healthcare professionals to ask about their problems and spend more time getting to the root of their problems [42]. Our study suggests that increased vigilance is needed when dealing with medically unexplained symptoms that may be psychosomatic manifestations of being bullied, particularly in girls, and in managing unexplained behavioural difficulties, especially in boys. In these instances, sensitive questioning about bullying may lead to disclosure and appropriate management, especially if referral is being considered. Our findings need to be replicated in the cohort as it moves forward and in other longitudinal studies. Further research is required to understand gender differences in health impacts of victimisation.

## 5. Conclusions

This study found that bully victimization as reported by children themselves is itself a significant factor in increasing the usage of healthcare services by children, having adjusted for the obvious effect of having a chronic condition and having free GP access. Different HCU patterns were observed according to gender and gender differences in the presentation of victimisation. We believe increased vigilance among health professionals is especially required when dealing with medically unexplained symptoms that may be psychosomatic manifestations of being bullied, particularly in girls, and in managing unexplained behavioural difficulties, especially in boys. At a wider population level, the development of clinical practice guidelines for the early detection and appropriate management of bullied children would help raise awareness among health professionals and parents about the impact of victimization on children’s health.

## Figures and Tables

**Table 1:** Proportion of being vicitimised by bullying and type of bullying in the past year by gender (total cohort N = 8183).

	Self-Report (Child)				Parent Report			
	All N (%)	Boys N (%)	Girls N (%)	** *p* value	All N (%)	Boys N (%)	Girls N (%)	** *p* value
Total Cohort	8183 (100)	4159 (100.0)	4024 (100.0)		8557 (100)	4375 (100.0)	4182 (100.0)	
Bully victim (all forms)	3265 (39.9)	1666 (40.1)	1599 (39.7)	0.77	2012 (23.5)	1009 (23.1)	1002 (24.0)	0.33
Physical	1630/2905 * (56.1)	1017 (67.2)	613 (44.1)	<0.001	775 (38.5)	533 (52.8)	242 (24.2)	<0.001
Verbal	2232/3001 * 74.4)	1220 (79.4)	1012 (69.1)	<0.001	1806 (89.8)	903 (89.5)	903 (90.1)	0.3
Electronic	141/2681 * (5.3)	73 (5.3)	68 (5.2)	0.9	50 (2.4)	19 (1.9)	31 (3.1)	0.07
Notes	386/2671 * (14.5)	179 (13.1)	207 (15.9)	0.04	91 (4.3)	19 (1.9)	72 (7.2)	<0.001
By exclusion	1833/2889 * (63.4)	870 (59.2)	963 (67.8)	<0.001	737 (35.1)	326 (32.3)	411 (41.1)	<0.001
Other	363 /3265 (11.1)	184 (11.0)	179 (11.2)	0.89	39 (1.9)	13 (1.3)	26 (2.6)	0.02

* missing data. ** *p* value is based on the chi squared test statistic examining difference in types of bullying by gender.

**Table 2:** Univariate analysis of health care utilisation, child health and socio-demographic characteristics in victimised and non-victimised cohorts (total cohort N = 8183).

	Victimised CohortN = 3265 (39.9%)		Non-Victimised CohortN = 4198 (60.1%)		Total CohortN = 8183	*p* Value
	N	%	N	%	N	
**HCU Within previous 12 months**						
**Contact with GP**						
Yes	1402	41.7	1958	58.3	3360	
No	1863	38.6	2960	61.4	4823	0.005
**Contact with another medical doctor**						
Yes	535	41.2	764	58.8	1299	
No	2729	39.7	4151	60.3	6880	0.3
**Contact with Mental Health Professional (MHP)**						
Yes	298	49.0	310	51.0	608	
No	2967	39.2	4608	60.8	7575	<0.001
**Visits to Emergency Department**						
Yes	497	40.7	724	59.3	1221	
No	2768	39.8	4194	60.2	6962	0.5
**At least 1 night in hospital over lifetime**						
Yes	1473	41.8	2052	51.2	3525	
No	1792	38.5	2866	61.5	4658	0.002
**Child factors**						
**Gender**						
Female	1599	39.7	2425	60.3	4024	
Male	1666	40.1	2493	59.9	4159	0.8
**Chronic Illness/disability**						
No	2864	43.6	3710	56.4	6574	
Yes	401	45.1	488	54.9	889	0.001
**Learning Disability**						
No	2227	38.5	3561	61.5	5788	
Yes	878	44.3	1102	55.7	1980	<0.001
**Ongoing chronic illness/disability and/or learning disability**						
No	667	45.3	805	54.7	1472	
Yes	2598	61.6	1620	38.4	4218	<0.001
**Childhood overweight/obese**						
No	2227	38.5	3561	61.5	5788	
Yes	878	44.3	1102	55.7	1980	<0.001
Parent/family factors						
**Level of Education primary caregiver**						
Degree primary or post grad.	559	39.3	863	60.7	1422	
Non Degree	544	41.8	758	58.2	1302	
Higher secondary /Tech/Vocational	1160	38.4	1862	61.6	3022	
Lower secondary	759	39.6	1160	60.4	1919	
None/Primary	243	46.9	275	5.3	518	0.003
**Household type**						
Couple 1-2 children	1185	41.3	1683	58.7	2868	<0.001
Couple 3+ children	1426	37	2429	63	3855	
Single 1-2 children	427	46.4	492	53.5	919	
Single 3+children	492	90.8	315	58.1	542	
**Median age primary caregiver (years)**	39		40		NA	
**State paid medical service access**						
No	2143	37.6	3549	62.4	5692	
Yes full	1033	45.9	1218	54.1	2251	<0.001
Yes GP only	89	37.2	150	62.8	239	
**Social Welfare recipient**						
No	2254	38.2	3648	61.8	5902	
Yes	1009	44.4	1265	55.6	2274	<0.001

**Table 3:** Logistic regression models of bully victimisation and HCU.

	GP VISITS Any Visits vs. None ^+^R^2^ = 0.048		MHP VISITS Any Visits vs. None ^+^R^2^ = 0.31		NIGHTS IN HOSPITAL Any Nights vs. None ^+^R^2^ = 0.04		ED VISITS Any Visits vs. None ^+^R^2^ = 0.03	
**Characteristic**	Adjusted OR (95% CI)	*p* value	Adjusted OR (95% CI)	*p* value	Adjusted OR (95% CI)	*p* value	Adjusted OR (95% CI)	*p* value
**Victimised by Bullying**								
No	ref							
Yes	1.13 (1.03–1.25)	0.02	1.31 (1.05–1.63)	0.02	1.07 (0.97–1.18)	0.18	0.99 (0.87–1.13)	0.88
**State paid medical service access**								
No	ref		Not in final model*	>0.05				
Yes full	1.42 (1.24–1.62)	<0.001			0.97 (0.84–1.13)	0.72	1.29 (1.1–1.52)	0.002
Yes GP only	1.18 (0.89–1.58)	0.26			1.01 (0.76–1.35)	0.94	0.7 (0.45–1.09)	0.12
**Gender**								
Female	ref							
Male	0.84 (0.76–0.93)	0.001	1.25 (0.99–1.56)	0.057	1.36 (1.23–1.50)	<0.001	1.23 (1.08–1.4)	0.004
**Chronic Illness/disability**								
No	ref							
Yes	2.27(1.92–2.69)	<0.001	3.45 (2.67–4.46)	<0.001	2.28 (1.92–2.70)	<0.001	1.47 (1.21–1.78)	<0.001
**Learning Disability**								
No	ref							
Yes	1.43 (1.21–1.7)	<0.001	16.6 (13.2–21.0)	<0.001	0.98 (0.97–0.99)	<0.001	0.97 (0.96–0.98)	<0.001
**Household type**								
Couple 1-2 children	ref							
Couple 3+ children	0.69 (0.63–0.77)	<0.001	0.97 (0.76–1.23)	0.80	0.81 (0.73–0.90)	<0.001	0.76 (0.66–0.88)	<0.001
Single 1-2 children	1.08 (0.88–1.32)	0.46	1.75 (1.17–2.60)	0.006	0.93 (0.75–1.13)	0.45	0.92 (0.74–1.2)	0.92
Single 3+children	0.63 (0.46–0.87)	0.005	1.57 (0.85–2.88)	0.15	0.81 (0.59–1.11)	0.19	0.79 (0.6–1.04)	0.79
**Level of Education**								
Degree (primary or post grad.)	ref							
Non Degree	0.85 (0.72–1.0)	0.05	0.61 (0.42–0.89)	0.009	1.11 (0.94–1.31)	0.23	0.83 (0.66–1.03)	0.09
Non Degree	0.85 (0.72–1.0)	0.05	0.61 (0.42–0.89)	0.009	1.11 (0.94–1.31)	0.23	0.83 (0.66–1.03)	0.09
Higher secondary /Tech/Vocational	0.76 (0.66–0.87)	<0.001	0.65 (0.48–0.88)	0.005	1.10 (0.96–1.28)	0.18	0.74 (0.61–0.89)	0.001
Lower secondary	0.68 (0.58–0.79)	<0.001	0.43 (0.30–0.61)	<0.001	0.98 (0.84–1.18)	0.97	0.93 (0.76–1.14)	0.49
None/Primary	0.75 (0.58–0.98)	0.04	0.10 (0.05–0.22)	<0.001	1.04 (0.79–1.37)	0.76	0.98 (0.73–1.31)	0.88
**Childhood overweight/obesity**								
No	ref							
Yes	Not in final model *	>0.05	Not in final model *	>0.05	Not in final model ^*^	>0.05	Not in final model *	>0.05
**Social Welfare recipient**								
No	ref							
Yes	Not in final model ^a^	>0.05	Not in final model	>0.05	1.27 (1.11–1.47)	0.001	Not in final model ^a^	>0.05

* Fully adjusted backwards stepwise logisitic regression model. +Neglekirke R**^2^**. MHP = Mental Health Practitioner. ED = Emergency Department. Ref: Reference level of factor variable.

**Table 4:** Poisson Regression Models showing independent effect of victimisation on HCU.

	Beta Co-Efficient	IRR	Std Error	95% CI	*p* Value
**GP Visits**					
All	0.103	1.11	0.026	1.05–1.16	<0.001
Boys	0.057	1.06	0.039	0.98–1.14	0.14
Girls	0.134	1.14	0.036	1.06–1.23	<0.001
**MHP Visits**					
**Model 1 a**					
All	0.143	1.15	0.068	1.01–1.32	0.04
Boys	0.208	1.23	0.087	1.04–1.46	0.02
Girls	0.038	1.04	0.110	0.83–1.29	0.73
**Model 2 b**					
All	0.125	1.13	0.068	0.99–1.29	0.07
Boys	0.193	1.21	0.088	1.02–1.44	0.03
Girls	0.017	1.02	0.110	0.82–1.26	0.88
**Nights in hospital**					
**Model 1 a**					
All	0.072	1.08	0.017	1.04–1.11	<0.001
Boys	0.035	1.04	0.023	0.99–1.08	0.13
Girls	0.110	1.12	0.025	1.06-1.17	<0.001
**Model 2 c**					
All	0.057	1.06	0.017	1.02–1.09	0.001
Boys	0.026	1.03	0.023	0.98–1.07	0.27
Girls	0.093	1.10	0.025	1.04–1.15	<0.001

a: Adjusted for gender, chronic illness, learning disability, medical card, social welfare entitlement, social class household type, childhood overweight /obesity; b: adjusted for above and visits to GP. c: adjusted for above, visits to GP and visits to MHP (Mental Health Practicitioner).

## Abbreviations

GUI: Growing Up in Ireland, HCU: Healthcare Utilisation, MHP: Mental Health Professional, LD: Learning Disability.
